# Development of a clinical trial to determine whether watchful waiting is an acceptable alternative to surgical repair for patients with oligosymptomatic incisional hernia: study protocol for a randomized controlled trial

**DOI:** 10.1186/1745-6215-13-14

**Published:** 2012-02-07

**Authors:** Johannes C Lauscher, Peter Martus, Andrea Stroux, Jens Neudecker, Uwe Behrens, Ralf Hammerich, Heinz J Buhr, Jörg-Peter Ritz

**Affiliations:** 1 Department of General, Vascular, and Thoracic Surgery, Charité Campus Benjamin Franklin, Hindenburgdamm 30, 12200 Berlin, Germany; 2 Institute of Biometry and Clinical Epidemiology, Charité Campus Mitte, Charitéplatz 1, 10117 Berlin, Germany; 3 Department of General, Viszeral, and Thoracic Surgery, Charité Campus Mitte, Chirurgie, Germany; 4 Coordinating Center for Clinical Studies, Charité Campus Virchow Klinikum, Augustenburger Platz 1, 13353 Berlin, Charité Campus Mitte, Hindenburgdamm 30, 12200 Berlin, Germany; 5 Division of Medical Controlling, Charité Campus Mitte, Charitéplatz 1, 10117 Berlin, Germany

**Keywords:** oligosymptomatic incisional hernia, watchful waiting, surgical repair, prospective randomized trial

## Abstract

**Background:**

Incisional hernia is a frequent complication in abdominal surgery. This article describes the development of a prospective randomized clinical trial designed to determine whether watchful waiting is an acceptable alternative to surgical repair for patients with oligosymptomatic incisional hernia.

**Methods/Design:**

This clinical multicenter trial has been designed to compare watchful waiting and surgical repair for patients with oligosymptomatic incisional hernia. Participants are randomized to watchful waiting or surgery and followed up for two years. The primary efficacy endpoint is pain/discomfort during normal activities as a result of the hernia or hernia repair two years after enrolment, as measured by the hernia-specific Surgical Pain Scales (SPS). The target sample size of six hundred thirty-six patients was calculated to detect non-inferiority of the experimental intervention (watchful waiting) in the primary endpoint. Sixteen surgical centers will take part in the study and have submitted their declaration of commitment giving the estimated number of participating patients per year. A three-person data safety monitoring board will meet annually to monitor and supervise the trial.

**Discussion:**

To date, we could find no published data on the natural course of incisional hernias. To our knowledge, watchful waiting has never been compared to standard surgical repair as a treatment option for incisional hernias. A trial to compare the outcome of the two approaches in patients with oligosymptomatic incisional hernias is urgently needed to provide data that can facilitate the choice between treatment options. If watchful waiting was equal to surgical repair, the high costs of surgical repair could be saved. The design for such a trial is described here.

This multicenter trial will be funded by the German Research Foundation (DFG). The ethics committee of the Charité has approved the study protocol. Approval has been obtained from ten study sites at time of this submission. The electronic Case Report Forms have been created. The first patient was to be randomized November 14^th^, 2011. An initiation meeting took place in Berlin January 9^th^, 2012.

**Trial Registration:**

ClinicalTrials.gov: NCT01349400

## Background

Incisional hernias occur in 10-20% after elective abdominal incisions and are among the most frequent surgical complications after laparotomy [1]. Forty-four thousand incisional hernia repairs were performed in Germany in 2007 [2]. The treatment of incisional hernias thus represents a large surgical and socioeconomic problem.

A 2009 Danish study addressing the early postoperative outcome reported a major complication rate of 3.5%, an overall morbidity rate of 10.7%, and a mortality rate of 0.4%. The rehospitalization rate was 11.2% [3]. The long-term outcome of incisional hernia repair is even more unsatisfactory. Pain persists in 20% of the patients after hernia repair, and the recurrence rate after incisional hernia repair remains high despite the development of various tension-free techniques (10-50%) [4]. Since incisional hernia repair is not a low-risk operation, it should be performed only when definitely indicated.

Due to the risk of incarceration, surgery has thus far been regarded as the only reasonable therapy for patients with incisional hernia. An incarceration is defined as an acute retention of the hernia accompanied by acute severe pain. If not reduced or repaired immediately, the acute incarcerated hernia leads to impairment of perfusion of the hernal contents followed by necrosis. Acute incarceration is a serious complication associated with high mortality, which is significantly increased if resection of gangrenous bowel is required [5]. Other potential factors favoring surgical repair are enlargement of hernia with the consequence of more difficult repair and cosmetic reasons.

In fact, there have been no investigations concerning the actual risk of incarceration or risk factors for incarceration in the total population at risk. Some smaller studies have reported acute incarceration as the indication for surgery in 6.0 to 14.6% of incisional hernia repairs [6-9]. A pre-study in which we prospectively enrolled all patients with incisional hernia repair disclosed an emergency repair rate of 3.2% [10].

In a large prospective randomized trial, Fitzgibbons et al. demonstrated that the acute incarceration rate is low in inguinal hernias: 1.8 per 1000 patient-years in mildly symptomatic men [11]. The percentage of patients with pain and discomfort interfering with normal activities did not differ between watchful waiting and surgical repair after a 2-year follow-up [11]. Delaying surgical repair was found to have no adverse effect on subsequent surgery or the final outcome [12].

Besides medical considerations, health care cost containment is gaining increasing importance, and the cost-effectiveness of each procedure has to be taken into account. A comparison of tension-free repair and watchful waiting in men with minimally symptomatic inguinal hernias showed $1831 higher mean costs per patient for tension-free repair after a 2-year follow-up [13].

These randomized controlled trials indicate that watchful waiting should be considered in men with asymptomatic or minimally symptomatic inguinal hernia according to the European Hernia Society Guidelines, level of evidence 1B [14].

Many surgeons believe that repair of incisional hernias may become more difficult in the course of time due to hernia enlargement. Also, there is a lack of data on the natural course of incisional hernias (enlargement, pain and discomfort) and the subjective disturbance by the hernia for cosmetic reasons.

A relevant proportion of incisional hernia patients are already left without surgical correction, although there are virtually no data describing or predicting the natural course. Twenty-two percent of patients presenting with incisional hernia to outpatient departments of internationally renowned hernia surgeons received no surgical treatment. The percentage of patients submitted to surgical repair varied from 50 to 100% for different surgeons [15]. Obviously, the treatment of incisional hernias is not standardized even in experienced hands.

Due to evidence-based data, watchful waiting has become a reasonable option for minimally symptomatic inguinal hernias. The potential benefit of a watchful waiting strategy is the avoidance of a possibly unnecessary procedure with all its complications (e.g., wound infection, mesh infection, bowel injury, ileus, postoperative bleeding, recurrence, chronic postoperative pain) and cost containment. A randomized controlled trial comparing watchful waiting and surgical repair in oligosymptomatic incisional hernias is urgently needed to broaden our knowledge regarding the natural course of incisional hernias and to test watchful waiting as a treatment option.

## Methods/Design

### Objectives, endpoints and sample size calculation

This clinical trial is designed to investigate the hypothesis that watchful waiting is not inferior to surgical repair of asymptomatic and oligosymptomatic incisional hernias in terms of pain and discomfort during normal activities.

To rule out published or ongoing trials dealing with the same subject, a literature search was done. Searching PubMed using the terms "incisional hernia AND watchful waiting" retrieved no matching results. "Incisional hernia AND randomized trial" retrieved 180 hits, but no randomized trial comparing watchful waiting to surgical repair. By searching relevant websites http://www.clinicaltrials.gov, http://www.actr.org.au, http://www.umin.jp/ctr/index.htm, http://www.trialregister.nl, http://isrctn.org, no randomized trial comparing watchful waiting vs. surgical repair in incisional hernia could be identified.

The primary efficacy endpoint is pain/discomfort during normal activities as a result of the hernia or hernia repair 2 years after enrolment, as measured by the hernia-specific Surgical Pain Scales (SPS). Studies show that a long term follow up is needed because postoperative pain may decrease with time [16]. Participants are asked to rate the average pain during the last 24 hours on a 150 mm visual analog scale varying from "no pain sensation" to "most intense pain imaginable". The Surgical Pain Scales were introduced by McCarthy et al. Intraclass correlation coefficients for SPS were 0.95 and 0.94. Correlations varying from 0.44 to 0.60 between the visual analog scales and the bodily pain dimension on the SF-36 and significant differences between SPS levels for patients requiring more and less time to resume normal activities (p = 0.015 to p = 0.002) supported the validity of the scales [17]. SPS scores are measured at baseline and at follow-up visits after 6, 12, 18 and 24 months.

The sample size calculation is based on the non-inferiority of the experimental intervention. A mean SPS score of 12.0 with a SD of 12.0 and 12.0 and an expected mean difference of 0.5 in favor of the surgical intervention group is assumed after a two-year follow-up.

Thus, if both groups have a sample size of 286, a two-group 0.05 level one-sided t-test will have 80% power to reject the null hypothesis that watchful waiting is not equivalent to surgical repair in favor of the alternative that watchful waiting is not inferior to surgical intervention [18]. This leads to a total sample size of n = 2 × 286 = 572. Assuming a drop-out rate of about 10%, 636 patients will have to be allocated. Sample size calculation was done with nQuery 6.0.

The most important secondary endpoint (S1) is the cost of treatment. Hospitalization costs can be calculated yearly along the guidelines of the InEK (Institute for Hospital Reimbursement System) institute. Hospitalization costs in Germany are mapped by the German-Diagnosis Related Groups-System. Inpatient costs for each participant (operation; medical staff and nursing staff on ICU, on IMC and on general ward; medication; implants; bandaging and other material; catering; infrastructure; laboratory, radiological, endoscopic and cardiologic diagnostics) will be completely included. Hence, the exact costs of every patient can be calculated in Euro [19]. Indirect costs (time off from work) will be monitored by questionnaire during follow-up.

If the results show a statistical difference in the primary endpoint, S1 will also be tested for significance (hierarchical testing). We hypothesize lower costs in the watchful waiting group, and, if a statistical difference is found, patient satisfaction with care will be tested as another secondary endpoint S2. We also hypothesize superiority of the experimental intervention in the secondary endpoint S2 because surgery is avoided.

Additional secondary outcomes include pain at rest, pain during exercise, highest level of pain measured by the SPS [17]. The impairment of daily activities due to pain is measured by the German version of the Pain Disability Index (PDI). The German version of the PDI revealed objectivity, reliability (Cronbach's α 0.90) and factor and construct validity [20]. Principal axis factor analysis has been used to investigate the underlying dimensions of the PDI; parallel analysis and scree plot favored a single-factor solution [21]. The functional status and quality of life is measured by the 2^nd ^German version of the SF-36 questionnaire. The reliability of the German version of the SF-36 is satisfactory, the validity is adequate, and the correlation with another established quality of life questionnaire (Nottingham-Health-Profile) is high [22]. Depression/anxiety is assessed by the 3^rd ^German version of the Hospital Anxiety and Depression Scale (HADS-D). Sensitivity and specificity were both 0.80. Reliability is high (Cronbach's α 0.80) [23]. Postoperative complications (e.g., postoperative bleeding, hematoma/seroma, wound infection, mesh infection, intra-abdominal abscess, ileus, bowel obstruction, enterocutaneous fistula, bowel injury) and mortality are recorded.

The relative frequency of acute hernia incarceration (acute incarceration events/duration of hernia) is determined in the experimental group according to the Fitzgibbons' trial in inguinal hernia [24]. To document the natural course of the hernia and a potential enlargement, hernia size is measured by ultrasound in the watchful waiting group at enrolment and after a 2-year follow-up. Therefore, ultrasound of the abdominal wall is performed and the maximum diameter of the hernia is also determined. The recurrence rate is calculated in the control group.

**Figure 1:**
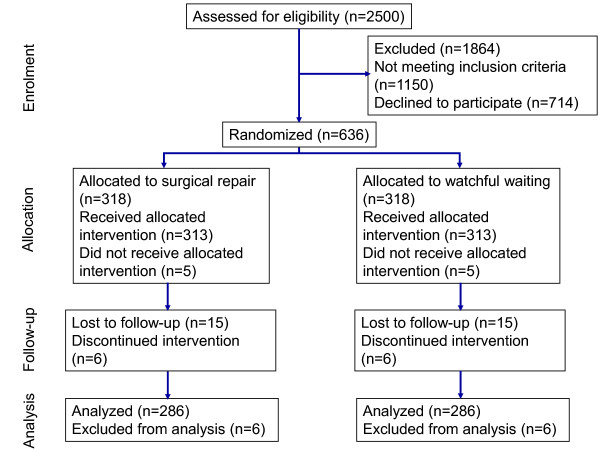
**Estimated trial flow of the study**.

A flow chart of the estimated number of screened patients and the number of randomized and analyzed patients according to the CONSORT 2010 statement for reporting parallel group randomized trials [25] is given in Figure 1.

### Inclusion and exclusion criteria

Inclusion and exclusion criteria were chosen broadly to provide generabilizability. Patients are eligible for enrolment if they meet both of the following criteria: 1) age ≥ 18 years, 2) asymptomatic/oligosymptomatic incisional hernia (patients with recurrent incisional hernias are eligible).

Patients are excluded if they meet any one of the following criteria: 1) no hernia detectable on physical examination, 2) acute incarcerated hernia, 3) emergency hernia repair, 4) hernia-related pain or discomfort during normal activities, 5) local or systemic infection, 6) ASA score > 3, 7) inability to complete or comprehend the preoperative questionnaire, 8) repair with biologic prosthesis.

### Implementation of the study

All patients presenting to the outpatient clinic with a possible incisional hernia are screened for trial eligibility. Those who have no unequivocal contraindications are scheduled to see the participating surgeon to confirm that they are candidates for the study. Informed consent is obtained for participation and randomization after taking a thorough history, performing a physical examination and checking for inclusion and exclusion criteria. Randomization will be done by an electronic randomization tool created by the Coordinating Center for Clinical Studies (KKS Charité). After obtaining the patient's informed consent, the site coordinator will log into the password-protected electronic randomization tool, which provides stepwise explanations. Randomization will be stratified by study center, hernia size (< 4 cm vs. ≥ 4 cm) and planned surgical repair (laparoscopic vs. open).

In primary analysis, adjustment for strata will be performed. We will additionally perform subgroup analyses within the strata and present the individual 95%-CIs. Furthermore, strata specific effect size calculations are planned if necessary.

Considering the lack of a prospective study on incisional hernia repair and the variety of repair techniques, many different methods can be adopted to enable generalizability and adequate recruitment.

Open incisional hernia repair will be performed with mesh (non-absorbable or partly absorbable alloplastic material) or sutures. Laparoscopic repair will be performed with non-absorbable or partly absorbable mesh in intraperitoneal onlay position (IPOM). The surgeon performing the operation can choose the optimal approach. Sublay mesh repair with closure of the peritoneum is recommended for hernias measuring ≥ 3 cm. IPOM, inlay or onlay repair are also permitted. The mesh should widely overlap the fascial margin (≥ 4 cm) on all sides. In non-mesh repair, either direct suture repair or the Mayo procedure [26] can be performed. The type of repair, the type of mesh, the suture material, the suturing method, and the peritoneal and fascial closure have to be documented to identify potential differences by subgroup analysis.

Participants randomized into the watchful waiting group will receive standardized oral information and written instructions which, like those described by Fitzgibbons et al., will deal with physical activity, diet, pain and pain medication, constipation management, sexual activity, hernia warning signs, and symptoms of acute incarceration [24]. They will be told to consult a physician immediately if acute symptoms develop. Physical examinations will be performed during follow-up visits at 1 month, 12 months and 24 months by a physician. After 6 and 18 months, participants will also be interviewed regarding potential hernia-related pain or discomfort by a physician or a study nurse. The frequency and scope of study visits is presented in table 1.

**Table 1:** Frequency and scope of study visits

Visit	Visit 1 (Screening)	Visit 2 (Day of surgery)	Visit 3 (1 month post OP)	Visit 4 (6 months post OP)	Visit 5 (12 months post OP)	Visit 6 (18 months post OP)	Visit 7 (24 months post OP)
Demographics and baseline clinical data	X						

Inclusion/exclusion criteria	X						

Randomization	X						

Clinical examination	X		X		X		X

Ultrasound	X						X

Pain level (SPS, PDI)	X			X	X	X	X

Quality of life (SF-36)	X			X	X	X	X

Mental health (HADS-D)	X						X

Patient satisfaction (Likert scale)	X			X	X	X	X

Surgical intervention		X					

Postoperative complications			X		X		X

AEs/SAEs		X	X	X	X	X	X

Costs of treatment		X	X	X	X	X	X

### Monitoring and statistical analysis

The Coordinating Center for Clinical Studies (KKS Charité) deals with project management, monitoring, and data management including randomization. After assessing the trial sites for qualification during a pre-study visit, the monitor will visit them on a regular basis. Visits will take place at least as follows: prior to enrolment of the first patient, three times during the course of the study, and at the time of study completion. Monitors work according to the Standard Operating Procedures (SOPs) of the KKS.

The following adverse events (AEs) are monitored (underlined): A wound infection is defined as a purulent discharge from the wound or detection of bacteria in wound samples taken using aseptic technique [27]. An intra-abdominal abscess must meet a least one of the following criteria: purulent drainage from a drain which is placed into the abdomen; organisms isolated from intra-abdominal aseptically obtained culture; abscess verified by direct examination during operation or by radiologic examination [27]. A mesh infection is defined as a detection of bacteria on the mesh with local signs of infection (redness, hyperthermia, swelling and pain) or general signs of infection like fever or tachycardia without any other reason [28]. Sepsis is recognized clinically as the presence of two or more of the following: 1) temperature greater than 38.5°C or less than 35.0°C; 2) heart rate greater than 90 bpm; 3) Respiratory rate greater than 20 bpm or PaCO2 < 32 mmHg; 4) WBC count greater than 12,000 cells/mm^3 ^or less than 4000 cells/mm^3 ^and an association with infection confirmed by culture or strongly suspected [29]. Intestinal injury or organ injury is defined as the transmural injury or the intestinal/organ wall. An enterocutaneous fistula is defined as a direct connection from the intestines to the skin with discharge of stools through the fistula.

Postoperative bleeding is defined as a bleeding requiring either blood transfusion or reoperation. Pneumonia is defined as an infection of the lung that can be caused by nearly any class of organism (bacteria, amoebae, viruses, fungi, and parasites) known to cause human infections. A urinary tract infection is an infection of one or more structures in the urinary system. A deep vein thrombosis is a blood clot in a major vein that usually develops in the legs or pelvis. Pulmonary embolism is an obstruction of a blood vessel in the lungs, usually due to a blood clot. A bowel obstruction is defined as the necessity to insert a nasogastric tube longer than 72 h after the operation or the necessity to reoperate. A recurrent hernia is a hernia diagnosed by physical examination or ultrasound after surgical hernia repair.

Other AEs are: vomiting and pain at rest. The following severe adverse events (SAEs) are monitored: reoperation, rehospitalization, ICU admission, acute incarceration, death. The principal investigator is informed about SAEs within 3 days.

An independent Data Safety Monitoring Board (DSMB) consisting of 3 external experts (2 clinical experts and 1 statistician) will meet annually to address patient safety and perform risk-benefit assessments. In accordance with its standard operating procedures (SOPs), the DSMB reviews the accumulating data from the ongoing trial to ensure continued patient safety. The DSMB assesses study aspects such as progress, integrity, and design and makes recommendations to the coordinating investigator regarding modification, continuation or termination of the study.

The primary statistical analyses will be conducted by the Institute of Biometry and Epidemiology at the Charité using an intention-to-treat (ITT) approach. Interim analysis is not planned. Additional as-treated analyses will be performed. Investigators are masked to the patient's treatment arm at the time of enrolment. The statistician will be blinded while performing data analyses.

## Discussion

This article describes the design of a trial comparing surgical and nonsurgical management of incisional hernias. Incisional hernia repair is not a low-risk operation, and it is associated with a high recurrence rate and a high percentage of postoperative pain. Treatment of incisional hernias represents a large surgical and socioeconomic problem. Up to now, surgical treatment has been recommended for incisional hernia patients regardless of whether there are any symptoms indicating a high risk of acute incarceration with serious complications. No studies have as yet defined the exact indications for incisional hernia repair or described the natural course of an incisional hernia, including the risk of acute incarceration. Randomized controlled trials performed in the past few years have shown that watchful waiting is a reasonable option for mildly symptomatic inguinal hernias.

To our knowledge, this is the first study to compare watchful waiting with surgical repair of oligosymptomatic incisional hernias in a prospective randomized setting. The study will provide very useful information regarding the natural course of incisional hernias and the rate of incarceration. We hypothesize that pain intensity during everyday activities does not differ between the compared groups and that the incarceration rate is low. If this should be confirmed, it would be justifiable to apply a watchful waiting strategy for oligosymptomatic incisional hernias and to thus avoid the risks and costs of surgery.

### Trial status

AWARE is funded by the German Research Foundation (DFG; project funding reference number LA 2380/3-1). Thus adequate financial resources are available to hire appropriate personnel (e.g., research nurses, project managers, monitors, and data managers) and cover additional expenses.

The study protocol has been approved by the Ethics Committee of the Charité. The trial protocol was registered http://clinicaltrials.gov/ on May 05, 2011 and was given a unique number for a worldwide identification of this trial (identifier: NCT01349400).

All 19 study sites received a study synopsis and signed the Declaration of Commitment. Participating study sites are: Charité Campus Benjamin Franklin, Berlin; Charité Campus Mitte, Berlin; Charité Campus Virchow Klinikum, Berlin; Universitätsklinikum Heidelberg; Universitätsklinikum Freiburg; Universitätsklinikum Carl Gustav Carus Dresden; Medizinische Fakultät Mannheim; HELIOS Klinikum Erfurt; HELIOS Klinikum Schwerin; Klinikum Südstadt Rostock; Johannes Gutenberg Universität Mainz; Klinikum Augsburg; Universitätsklinikum Bonn; Krankenhaus Sinsheim; Universitätsklinikum Münster, Diakoniekrankenhaus Henriettenstiftung Hannover, Ludwig-Maximilians-Universität München, Unfallkrankenhaus Berlin, Klinikum rechts der Isar der Technischen Universität München.

This declaration specified the inclusion and exclusion criteria, and the trial sites used hospital data management systems to estimate the annual number of patients willing to participate. According to these declarations, 949 patients will be enrolled in 36 months. Each center plans to enroll 5 to 40 patients each year. Non-university and university medical centers are involved to increase the generalizability of the study. All participating centers are familiar with clinical studies. Eight of them belong to the Chir-Net Germany, a network of regional surgical centers funded by the Federal Ministry of Education and Research (BMBF) and devoted to the development and implementation of multicenter clinical trials. All study centers have received the final study protocol. In 10 centers, the approval of the local ethics committees has been obtained.

The electronic case report forms (eCRFs) and the randomization tool have been completed.

An initiation meeting in Berlin took place in January 9, 2012 to introduce the principal investigators and to familiarize the study centers with the details of screening, randomization, surgery, monitoring and follow-up. The whole study team was present. The first patient was randomized at Charité Campus Benjamin Franklin November 14, 2011.

The recruitment period is planned for 36 months (until October 2014); last-patient-out should be achieved in November 2016.

## Abbreviations

AE: Adverse Event; ASA: American Society of Anesthesiologists; AWARE: Watchful Waiting vs. Repair of Oligosymptomatic Incisional Hernias; BMBF: Bundesministerium für Bildung und Forschung (Federal Ministry of Education and Research); bpm: Beats per minute; CI: Confidence interval; CONSORT: Consolidated Standards of Reporting Trials; DFG: Deutsche Forschungsgemeinschaft (German Research Foundation); DSMB: Data Safety Monitoring Board; eCRF: Electronic Case Report Form; HADS-D: Hospital Anxiety and Depression Scale - Deutsche Version (German version); ICU: Intensive Care Unit; IMC: Intermediate Care Unit; InEK: Institut für das Entgeltsystem im Krankenhaus (Institute for Hospital Reimbursement System); IPOM: Intraperitoneal onlay mesh; ITT: Intention-to-treat; KKS: Koordinierungszentrum für Klinische Studien (Coordinating Center for Clinical Studies); OP: Operation; PDI: Pain Disability Index; SAE: Serious Adverse Event; SD: Standard deviation; SF-36: Short Form 36 Health Survey Questionnaire; SOP: Standard operating procedure; SPS: Surgical Pain Scales.

## Competing interests

All authors declare that they have no competing financial interests and no competing non-financial interests.

## Authors' contributions

The idea of trial, the concept of the study and the study design was created by JCL and J-PR. JCL wrote the successful application for the grant of the Deutsche Forschungsgemeinschaft. The acquisition of data (first randomized patients) was done by JCL. The biometrical analysis including power calculations was done by PM and AS. RH is responsible for the calculation of inpatient costs. UB is the project manager responsible for administration of the trial. JN as the head of the Surgical Regional Center Berlin is supporting the coordination of the trial. JCL has drafted the manuscript. The critical revision of the manuscript was done by JCL, PM, AS, JN, UB, RH, HJB, and J-PR. All authors approved the final manuscript.
